# The importance of specialized sleep investigations in children with a suprasellar tumor

**DOI:** 10.1007/s11102-020-01065-9

**Published:** 2020-07-20

**Authors:** J. van Schaik, S. Pillen, R. R. L. van Litsenburg, N. L. E. Vandenbussche, J. M. de Bont, A. Y. N. Schouten-van Meeteren, H. M. van Santen

**Affiliations:** 1 Department of Pediatric Endocrinology, Wilhelmina Children Hospital, University Medical Center Utrecht, Lundlaan 6, Utrecht, 3584 EA The Netherlands; 2 Department of Sleep Medicine, Kempenhaeghe Expertise Center for Epileptology, Sleep Medicine and Neurocognition, Heeze, The Netherlands; 3 Princess Máxima Center for Pediatric Oncology, Utrecht, The Netherlands; 4 Emma Children’s Hospital, Amsterdam UMC, Vrije Universiteit Amsterdam, Pediatric Oncology Cancer Center Amsterdam, Amsterdam, The Netherlands

**Keywords:** Childhood brain tumor survivors, Suprasellar tumors, Sleep disturbances, Increased daytime sleepiness, Sleep investigation

## Abstract

**Purpose:**

Disruption of sleep has great impact on quality of life. In children with a suprasellar tumor and hypothalamic-pituitary dysfunction, the circadian rhythm may be disturbed causing sleep problems. However, also other factors may influence sleep. Awareness of these different etiologies and careful history taking with appropriate additional diagnostics will aid in restoring sleep quality.

**Methods:**

We present the workup of 4 cases with a suprasellar tumor and disturbances of sleep initiation, sleep maintenance, and daytime sleepiness. In parallel, we developed a flowchart, to aid clinicians in the diagnostics of sleep problems in children after treatment for a (supra) sellar brain tumor.

**Results:**

All four patients, known with hypopituitarism, presented with sleep complaints and increased daytime sleepiness. In all four, the cause of sleep problems showed to be different. In the first case, sleep evaluation revealed a severe obstructive sleep apnea, whereupon nocturnal ventilation was started. The second case revealed poor sleep hygiene in combination with an obsessive compulsive disorder. Sleep hygiene was addressed and psychiatric consultation was offered. Dexamphetamine treatment was started to reduce her obsessive compulsive complaints. The third case showed a delayed sleep phase syndrome, which improved by educational support. The fourth case revealed a secondary organic hypersomnia for which modafinil treatment was started.

**Conclusion:**

Sleep disturbances in children with hypopituitarism due to a (supra) sellar tumor can have different entities which require specific therapy. Awareness of these different entities is important to enable appropriate counseling. Referral to an expertise sleep center may be advised, if standard educational support is insufficient.

## Introduction

Due to increased survival rates for children with a CNS tumor, the late effects of the tumor and its treatment are becoming more visible and more important [1]. Childhood brain tumor survivors (CBTS) often report a low quality of life and an important factor that may influence the degree of quality of life is their quality of sleep [2].

**Fig. 1:**
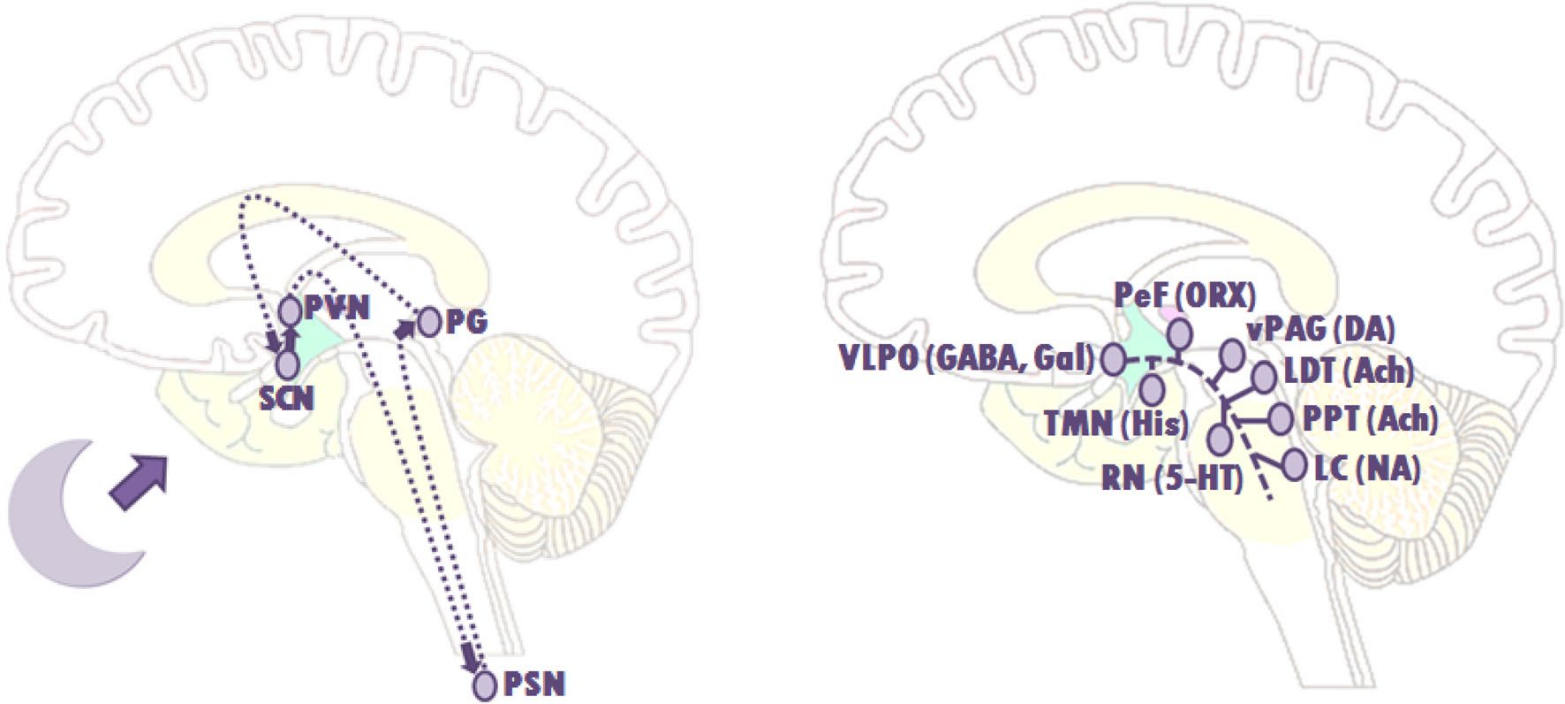
Schematic drawing of the circadian rhythm and arousal system involved in the flip-flop switch. **a** A schematic drawing of the suprachiasmatic nuclei (SCN) controlling the circadian rhythm during sleep. Through activation of the SCN via the axons of retinal ganglion cells running in the optic nerves (forming a retino-hypotalamic tract), the SCN sends a signal to the paraventricular nuclei (PVN) and preganglionic sympathetic neurons (PSN), which in their turn activate the pineal gland (PG) to produce melatonin. **b** A schematic drawing to show the basic route of the ventrolateral preoptic nucleus (VLPO) to the main elements of the ascending arousal system, involved in the flip-flop switch. The arousal system consist of monoaminergic cell groups (MCG) such as the tuberomammillary nucleus (TMN), the raphe nuclei (RN) and the locus coeruleus (LC). VLPO neurons also stimulate the neurons in the lateral hypothalamus, such as the perifornical (PeF) and orexin (ORX) neurons, and the cholinergic (ACh) cell groups; the pedunculopontine (PPT) and laterodorsal tegmental nuclei (LDT). During a wakeful state, the MNG inhibit the VLPO neurons, thereby activating the arousal system trough the MNG, and that of the ORX neurons and the PPT and LDT. During sleep, the firing of the VLPO neurons suppress the MNG. This also allows it to inhibit the orexin neurons, further preventing monoaminergic activation that might interrupt sleep. The direct inhibition between both groups, the VLPO and the monoaminergic cell groups, forms the flip-flop switch. 5-HT, serotonin; GABA, γ-aminobutyric acid; gal, galanin; NA, noradrenaline; His, histamine

Sleep is a complex neurophysiologic process. The suprachiasmatic nuclei are key regulators of the circadian rhythm, controlling sleep and wakefulness [3, 4]. Melatonin, excreted by the pineal gland in the dark, greatly influences this circadian rhythm (Fig. 1a) [4]. In children with a (supra) sellar tumor the circadian rhythm may be disturbed due to a distorted melatonin excretion or a distorted response to melatonin [5–7]. In addition to the suprachiasmatic nuclei, the ventrolateral preoptic nucleus (VLPO) together with the lateral hypothalamus area (LHA) (sleep-promoting), and monoaminergic cell groups (MCG) (the arousal system) also regulate sleep [8]. These sleep-promoting pathways and arousal pathways can mutually influence each other by the “flip-flop” switch (Fig. 1b) [9]. This interaction rapidly promotes the transition between waking and sleeping. Damage to the VLPO results in insomnia and damage to the LHA results in both sleep disruption as well as daytime hypersomnolence [10–13].

It is well known that patients with a (supra) sellar tumor are prone to develop sleep problems or daytime sleepiness [14, 15]. While the physical damage caused by the tumor or its treatment is a probable causal mechanism, other risk factors, such as psychological, behavioral, and social environmental should also be considered. Parenting strategies that include regular bedtime routines and consistent limit setting are required to develop healthy sleep habits, but may be a challenge to parents of a child with a life-threatening or chronic disease [16, 17].

There are currently no guidelines for assessment of sleep disturbances in this group. Early referral to a specialized sleep clinic may reveal the etiology of the sleep disturbance and will give direction to the proper treatment. The following cases illustrate the importance of specialized diagnostics to distinguish the different etiologies of sleep disturbances in children with a suprasellar tumor enabling proper treatment.

### Case 1

A 13 year-old girl, presented with frequent headaches during day and night, inability to fall asleep, difficulty maintaining sleep, and daytime sleepiness. She was diagnosed with craniopharyngioma at the age of six and underwent gross total resection. Magnetic Resonance Imaging (MRI) of the brain at follow-up showed absence of the pituitary stalk and damage of the anterior and posterior hypothalamic region with atrophic mammillary bodies, scored by the neuroradiologist as grade II, according to the Muller grading for hypothalamic damage [18]. After surgery, she developed panhypopituitarism and morbid obesity (Body Mass Index (BMI) Standard Deviation Score (SDS) of 5.1). Patient had been adequately hormonally supplemented for all pituitary deficiencies and used dextroamphetamine for her hypothalamic obesity. Her medical history further contained repeated surgery of her knees due to varus position. Due to her knee problems together with her morbid obesity, she was partially wheel chair bound.

Sleep history revealed long screen time before and during bed time, difficulty in sleep initiation and maintenance, and worrying during the night. There were no complaints of snoring, but a moaning sound was frequently heard by parents in the night.

Polysomnography (PSG) with capnography revealed a normal sleep latency with a recognizable cyclic sleep structure. Patient had a continuous tachypnea of 35- 40 times per minute, with frequently a typical breathing pattern in REM sleep, starting with a deep sigh following a long exhale in combination with a moaning sound, indicative of catathrenia. She had obstructive sleep apnea, with an apnea–hypopnea index of 19.4 (normal in children < 1/h), and an oxygenation-desaturation index (≥ 3%) of 33.8 (> 10 indicative for moderate to severe obstructive sleep apnea). Lowest oxygen level was 63%. Her carbon dioxide was normal during the day, but increased upon awakening (49.4 mmHg) (Table 1). The melatonin test showed no increase in melatonin concentration (Table 2). Patient was diagnosed with obstructive sleep apnea, sleep related hypoventilation and catathrenia according to the ICSD-3 criteria. Patient was referred for nocturnal home bi-level ventilation, with a inspiratory pressure support of 18 and external pressure support of 6, after which she experienced less symptoms of insomnia, less headaches and less daytime sleepiness.

**Table 1:** Sleep evaluation data of all four cases

Subjective sleep data*	Case 1	Case 2	Case 3	Case 4
TIB	10 h 17 min	9 h 47 min	11 h 49 min	8 h 39 min
Bed time	8.32 p.m	9.08 p.m	9.26 p.m	9.47 p.m
Rise time	6.49 a.m	6.55 a.m	9.15 a.m	6.26 a.m
TST	5 h 54 min	6 h 58 min	8 h 17 min	7 h 37 min
SOL	1 h 49 min	1 h 12 min	1 h 14 min	48 min
WASO	2 h 17 min	20 min	41 min	1 h 53 min
WASF	24 min	1 h 28 min	1 h 2 min	14 min
*Questionnaire*				
ESS	12	–	11	23
MSLT	–	–	–	2.7 min, 1 SOREMP
Polysomnography**			–	
TIB	487.0 min	573.0 min		737.0 min
TST	463.5 min	500 min		547.5 min
SOL	1.0 min	4.5 min		0 min, time until first REM 369 min
WASO	22.5 min	68.5 min		18.5 min
SE	95.2%	87.3%		74.3%
Sleep stages				
REM	3.8%	17.0%		29.8%
NREM 3	48.8%	17.4%		11.7%
AHI	19.4, obstr	1.1, obs & centr		2.6, obstr & centr
AI	0.0	0.1		0.3
PLMI	0.0	9.0		5.8
ODI (3%)	33.8	2.2		1.3
ODI (4%)	11.1	1.2		0.5
Mean CO2^ level:	43.0	46.0		–
Highest CO2 REM:	46.2	47.5		
Highest CO2 NREM:	47.2	47.5		
Blood gas^^ analysis after sleep:	7 a.m.: pH 7.35, pCO2 49.3, HCO3 26.5, BE 0.9 11 a.m.: pH 7.42, PCO2 36.8, HCO3 23.2, BE − 0.5			
Mean O2 level:	95%	94%		98%
Lowest O2 level:	63%	86%		91%
Time below 90%:	5.7 min	9.5 min		0 min

*TIB* time in bed, *TST* total sleep time, *SOL* sleep onset latency, *WASO* Wake after sleep onset, *WASF* wake after sleep offset, *ESS* Epworth sleepiness scale, *MSLT* Multiple sleep latency test, *SOREMP* sleep onset REMsleep period, *SE* sleep efficiency, *REM* rapid eye movement sleep, *NREM* non rapid eye movement sleep, *AHI* apnea hypopnea index, *obstr* obstructive apnea, *centr* central apnea, *AI* apnea index, *PLMI* periodic limb movements of sleep, *ODI* oxygen desaturation index, *CO2* carbon dioxide, *HCO3* Bicarbonate, *BE* Base excess

– = data is not available or test is not performed

^*^Subjective sleep data consisted of data of 1 week graphical sleep wake diary, data of case 4 consisted of actigraphy data due to incorrect sleep–wake diary

^**^The polysomnography consisted of an all night supervised video polysomnography performed according to the AASM (American academy of sleep medicine) criteria version 2.6, including: (1) 8 EEG- channels (F4-M1, C4-M1, O2-M1, with back-up F3-M2, C3-M2 and O1-M2); (2) 2 electroculographic channels; and (3) 4 surface electromyographic channels: 2 from the mentalis, one from the right anterior tibialis and one from the left anterior tibialis in lower limbs; and (4) 7 channels to monitor respiratory function: 1 for oximetry, 1 for transcutaneous carbondioxide 2 for oro-nasal airflow using a thermistor and nasal-cannula, 2 to record thoraco-abdominal movements by inductance plethysmography, and a microphone at the suprasternal notch to detect snoring. Transcutaneous carbon dioxide measurement was calibrated with capillary blood gas samples preceding the PSG night and at awakening. Sleep stages were visually scored for 30-s epochs according to the guidelines of the American Academy of Sleep Medicine (AASM) version 2.6. Scoring was performed by an experienced sleep technologist and later reviewed by an accredited sleep specialist

^CO2 presented as mmHg

^^Blood gas presented as pH, CO2 = mm Hg, O2 = mm Hg, HCO3 = mmol/L, BE = mmol/L

**Table 2:** Measured melatonin values of four cases

Time	Case 1	Case 2	Case 3	Case 4
12.00 pm	0.9			
6.00 pm	< 0.5	< 0.5		0.8
7.00 pm		1.7		0.9
8.00 pm	1.6	6.3 (*DLMO 7.30*)	0.5	< 0.5
9.00 pm	< 0.5	< 0.5^	0.5	< 0.5
10.00 pm			1.0	1.1 (*DLMO 10.13*)
11.00 pm			4.6 (*DLMO 10.50*)	14.2
12.00 pm			6.5	28.01
1.30 am	*	4.8		
6.00 am		8.0		1.2
7.00 am	*			> 50.0^
8.00 am				2.2
9.00 am	< 0.5			

Values are represented as pg/ml measured in saliva

^*^Not enough material for detection

^Suspected measurement error due to food intake (breakfast)

*DLMO* Dim Light Melatonin Onset (the estimated time at which salivary melatonin reaches 4 pg/ml)

### Case 2

A 12 year-old girl with complaints of waking up earlier than desired and daytime sleepiness was referred for sleep evaluation. She had been diagnosed with craniopharyngioma at age five. Patient underwent subtotal resection, complicated by a subdural hematoma and meningitis. After surgery, patient received cranial irradiation. MRI of the brain at follow-up, after surgery and irradiation, showed severe anterior and posterior hypothalamic damage behind the mammillary bodies, grade II according to the Muller grading of hypothalamic damage. She experienced rapid weight gain (BMI SDS 4.3) with obsessive compulsive mood disorder. Medical history showed no other relevant comorbidities. Patient received adequate hormonal supplementation for all pituitary deficiencies.

During the night, the mother occasionally heard snoring and apneas. The girl experienced early awakenings (4.00 a.m.) with a strong urge to search for food. This food seeking behavior was present both day and night. She was aware of her behavior, but could not control this strong urge. Sleepiness was present in the morning and evening, which resulted in falling asleep in public places, with extreme difficulty to be woken up.

Both the PSG with capnography and melatonin test (Tables 1 and 2) showed normal results, with no indication for a obstructive sleep apnea syndrome or a circadian rhythm disorder. Based on her sleep wake calendar and history taking, she met the criteria of the ICSD-3 for a chronic insomnia disorder, in which her obsessive compulsive disorder and strong urge for food in combination with poor sleep hygiene were the most important perpetuating factors. For the prominent obsessive compulsive behavior, she was referred for psychiatric consultation. Dextroamphetamine was started to reduce her obsessive compulsive complaints, also aiming to positively affect her hypothalamic obesity.

### Case 3

A 16 year-old girl with complaints of difficulty initiating sleep and daytime sleepiness was referred for sleep evaluation. She was treated at age 8 years for suprasellar germinoma with chemotherapy, neurosurgery, and radiotherapy. MRI of the brain at follow-up showed no hypothalamic damage, only damage in the sellar region, grade 0 according to the Muller grading of hypothalamic damage. She had been diagnosed with panhypopituitarism and obesity. Patient had been adequately hormonally supplemented for all pituitary deficiencies. Medical history showed no other relevant comorbidities.

Her sleep history showed relatively long screen time before sleeping and irregular and long time in bed (between 11 and 14 h), but with a normal sleep duration of over 8 h (Table 1). She had no complaints of waking up during the night, snoring, dry mouth, or nycturia. She frequently had headaches during the day.

A dim light melatonin onset test was performed and showed a normal but late increase of melatonin (Table 2). She met the ISCD-3 criteria for a delayed sleep phase syndrome diagnosis together with an inadequate sleep hygiene. She received education for improving her sleep hygiene, with special attention to a regular sleep wake rhythm, less time in bed, and avoidance of screen time before going to bed. With this advice, the sleep wake rhythm normalized and daytime complaints disappeared.

### Case 4

A 16 year old boy presented with complaints of difficulty initiating sleep and maintaining sleep, waking up earlier than desired, and daytime sleepiness. He had been diagnosed with pilocytic astrocytoma in the suprasellar region and neurofibromatosis type I at age 7. Therapy consisted of subsequent episodes of neurosurgery, chemotherapy, and irradiation. MRI of the brain at follow-up, after neurosurgery, chemotherapy, and irradiation, showed abnormalities due to Moya-Moya syndrome and neurofibromatosis, plus anterior and posterior hypothalamic damage behind the mammillary bodies (grade II Muller et al.) with residual cystic mass in the suprasellar region. He developed panhypopituitarism in combination with the syndrome of inappropriate antidiuretic hormone (SIADH) and weight gain. Due to Moyamoya syndrome, patient also developed transient ischemic attacks. Patient had been adequately hormonally supplemented for all pituitary deficiencies. Medical history showed no other relevant comorbidities.

Sleep history showed insomnia during the night consisting of sleep onset problems, prolonged night time awaking of several hours, and early morning awakening. He frequently left bed during the night, to secretly eat and drink. This food seeking behavior was present both day and night. Patient was aware of this behavior, but could not control this strong urge. He also tended to secretly watch TV when awake in the night. Parents had already received guidance by behavioral therapists to alter this behavior, but still had difficulties in addressing this behavior, because they were often not aware of his night time awakenings. During the day, he suffered from severe hypersomnolence with falling asleep at school, when watching TV or even during meals, resulting a very high Epworth sleepiness scale score of 23 out of 24 (normal < 11).

Actigraphy confirmed insomnia with prolonged sleep onset, early morning awakening, and frequent naps during the day. PSG showed a normal cyclic sleep structure, with normal sleep duration of more than 9 h. The multiple sleep latency test showed a short sleep latency of 2.7 min (normal > 8 min) and one sleep-onset REM period (SOREMP) (Table 1). A mildly delayed increase of melatonin concentration in the evening was seen (Table 2). These mixed results made it difficult to make a formal ICSD-3 diagnosis. Narcolepsy was ruled out based on the MSLT results in combination with absence of other narcolepsy symptoms, but secondary organic hypersomnia was suspected based on the severe daytime sleepiness. Treatment was based on a multidisciplinary symptom-based approach. Behavioral therapy was started to address the behavior of going out of bed in the night and improving sleep hygiene. Additionally, pharmacological treatment with modafinil was started. These measures improved both his daytime functioning as well stabilized his sleep rhythm with less night time awakening.

## Discussion

Sleep disturbances in children surviving a (supra) sellar tumor are common [19]. These cases illustrate that, also in children with tumors in this location, extended sleep evaluation may reveal different etiologies enabling individual treatment. The fact that sleep disturbances are not only associated with decreased quality of life, but also with obesity, makes it of special interest in this patient group, as they often also cope with (hypothalamic) obesity [20–24]. For these reasons, sleep is one of the important clinical domains on which a child surviving a suprasellar tumor should be evaluated. When the sleep disturbance is adequately addressed, BMI and quality of life during the day and night may be improved [25].

Symptoms of a sleep disturbance in children with a tumor in the suprasellar region that have been frequently described in literature are difficulty initiating sleep, maintaining sleep, waking up earlier than desired, but also increased daytime sleepiness [7, 19, 26]. Increased daytime sleepiness can be a result of disturbed circadian rhythm, reduced sleep, or of insufficient hormonal substitution. Sleep and the circadian rhythm is mainly regulated by hypothalamic structures, such as the suprachiasmatic region involved in the melatonin secretion, the median and ventrolateral preoptic areas involved in the flip-flop switch model, and the lateral/posterior nuclei, important for regulating wakefulness [3, 8, 27]. Destruction of these structures, either due to the tumor or its treatment, thus play an important role in the etiology of sleep disturbances in children surviving a tumor in the hypothalamic-pituitary (HP) region [10, 11, 13, 28–30].

Many children surviving tumors in the HP region suffer from obesity [31]. As obstructive sleep apnea is very common in obese patients (upon 60%), children with hypothalamic obesity and complaints of fatigue or daytime sleepiness should be evaluated for the presence of obstructive sleep apnea. The complications of sleep apnea can be very severe (e.g. cardiovascular and metabolic, growth retardation and reduced quality of life) [30, 32, 33].

Next to sleep apnea, the melatonin production may be altered due to damage of the SCN. Decreased nocturnal melatonin levels have been associated with increased daytime sleepiness, higher BMI, and hypothalamic tumor diagnosis [34]. In 10 adult obese patients after treatment for childhood craniopharyngioma, treatment with melatonin was found to be successful to decrease daytime sleepiness. Melatonin treatment may be useful in decreasing sleep onset latency and improving sleep total time, however some question its safety for use in children given the broad (side) effects on multiple developing systems and it fails to target long term maintaining factors that cause sleep disruption to persist [35, 36]. Medication such as melatonin should therefore only be considered after extended additional diagnostics in a specialized sleep clinic, in agreement with a somnologist.

Secondary narcolepsy was considered in the fourth case, which has been related to the flip-flop switch [37]. In a study by Muller et al., seven of ten suprasellar tumor patients were found to fulfill the criteria for secondary narcolepsy [34]. Narcolepsy in patients with hypothalamic lesions is most likely due to direct injury to the orexin-expressing neurons (located in the lateral hypothalamus), which are involved in the flip-flop switch [38, 39]. Although case four did not meet the criteria for narcolepsy, his MSLT was very short, fulfilling the criteria of hypersomnia due to a medical condition. This short MSLT time can be classified as severe sleepiness and may be a consequence of hypothalamic damage resulting in disturbance of the flip-flop switch [40]. Treatments given for narcolepsy, such as modafinil, dexamphetamine, methylphenidate, and other stimulants may, in these situations, be effective [41, 42].

Last, but not least important, psychological or psychiatric complaints, poor sleep hygiene, and inappropriate light exposure may cause sleep disturbance resulting in insomnia and increased daytime sleepiness. Worrying thoughts, anxiety, depression, and obsessive compulsive mood disorder, which is more frequently seen in children with hypothalamic damage, may severely influence sleep quality [43]. This warrants a broad and multidisciplinary approach when dealing with sleep problems in these children. Further, particularly in adolescents, delayed sleep phase disorder is common due to lengthen of the circadian rhythm, as the child becomes more social. Inadequate sleep hygiene includes those habits that enhance wakefulness and interrupt the sleep period, leading to a decrease in the quality of sleep and increased daytime sleepiness. Examples of these habits include engaging in stimulating activities near bedtime, using the bed for activities not related to sleep, inconsistent bedtimes, and inappropriate napping [44]. Treatment of these disorders is primarily educational, with strict sleep structure and restriction of screen time [45]. Restriction in screen time will lead to more mobility and more social behavior [46]. Especially, mobility and physical activity during the day can improve sleep quality drastically [47–49].

**Fig. 2:**
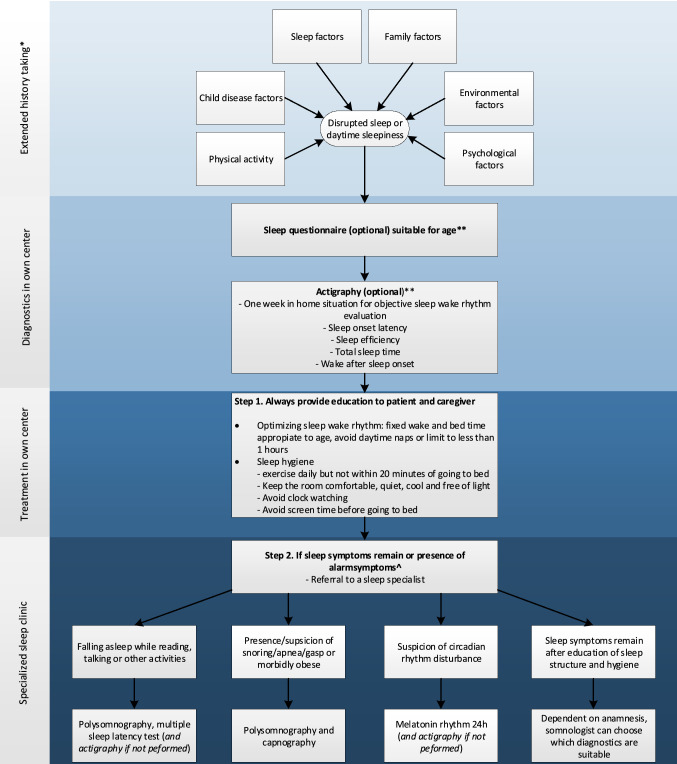
Proposed additional diagnostics regarding sleep disturbances in children with a brain tumor. *Extended history taking consists of: 1. Sleep factors: sleep wake pattern (sleep schedule, bedtime routine, screen time), total sleep time, nycturia, wake after sleep onset time, sleep symptoms (snoring, sleepwalking), daytime symptoms (morning headache, daytime sleepiness, naps). 2. Child disease factors: medical history, current treatment, pain, side-effect of treatment, medication. 3. Psychological factors: stress factors, anxiety, trauma, depression. 4. Family factors: parenting styles, parenting belief, attitude, siblings. 5. Environmental factors: sleep environment, sleep position. 6. Physical activity: transport, sport, school. **For examples and additional information of suitable sleep questionnaires or actigraphy, see: Table 1https://doi.org/10.1002/pon.5242 [50]. ^Alarm symptoms: falling a sleep during activities or snoring, apnea, or gasping during sleep

Of our four cases, all of them showed inadequate sleep hygiene, which needed to be addressed. Providing knowledge and guidance to parents about adequate sleep hygiene and structure for their child, during and after cancer treatment should therefore be considered as standard care and may play an important role in prevention of sleep disturbances. To aid in diagnostics of sleep problems in children after treatment for a (supra) sellar brain tumor, a flowchart is proposed (Fig. 2).

## Conclusion

Children surviving a (supra) sellar tumor may present with sleep disturbances due to variable causes. Education of sleep hygiene and bedtime structure is important for all. Referral to an expertise sleep center may help to distinguish the different causes of sleep problems in this specific patient group and enable adequate intervention.

## Data Availability

Deidentified individual participant data will not be made available.

## Abbreviations

CBTS: Childhood Brain Tumor Survivor
LHA: Lateral Hypothalamic Area
BMI: Body Mass Index
PSG: Polysomnography
Gray: Gy
ESS: Epworth Sleepiness score
SOREMP: Sleep-onset REM period
HP: Hypothalamic-pituitary
SCN: Suprachiasmatic nucleus
MSLT: Multiple Sleep Latency Test
